# Lenient rate control versus strict rate control for atrial fibrillation: a statistical analysis plan for the Danish Atrial Fibrillation (DanAF) randomized clinical trial

**DOI:** 10.1186/s13063-023-07247-7

**Published:** 2023-04-01

**Authors:** Isak Mazanti Cold, Joshua Buron Feinberg, Axel Brandes, Ulla Davidsen, Ulrik Dixen, Helena Dominguez, Uffe Jakob Ortved Gang, Christian Gluud, Rakin Hadad, Kit Engedal Kristensen, Doan Tuyet van Le, Emil Eik Nielsen, Michael Hecht Olsen, Ole Dyg Pedersen, Ilan Esra Raymond, Ahmad Sajadieh, Anne Merete Boas Soja, Janus Christian Jakobsen

**Affiliations:** 1 The Faculty of Health and Medical Sciences, The University of Copenhagen, Copenhagen, Denmark; 2 Copenhagen Trial Unit, Centre for Clinical Intervention Research, The Capital Region, Copenhagen University Hospital – Rigshospitalet, Copenhagen, Denmark; 3 Department of Internal Medicine – Section of Cardiology, Holbaek Hospital, Holbaek, Region Zealand Denmark; 4 Department of Regional Health Research, The Faculty of Health Sciences, University of Southern Denmark, Odense, Denmark; 5 Department of Cardiology, Odense University Hospital, Odense C, Denmark; 6 Department of Cardiology, Esbjerg Hospital – University Hospital of Southern Denmark, Esbjerg, Denmark; 7 Department of Clinical Research, Faculty of Health Sciences, University of Southern Denmark, Odense, Denmark; 8 Department of Cardiology, Copenhagen University Hospital Bispebjerg and Frederiksberg, Copenhagen, Capital Region of Denmark Denmark; 9 Department of Cardiology, University Hospital Amager and Hvidovre, Copenhagen, Denmark; 10 Department of Biomedicine, Faculty of Health and Medical Sciences, University of Copenhagen, Copenhagen, Denmark; 11 Department of Cardiology, Zealand University Hospital Roskilde, Roskilde, Region Zealand Denmark

**Keywords:** Atrial fibrillation, Rate control, Randomized trial, Statistical analysis plan

## Abstract

**Background:**

A key decision in the treatment of atrial fibrillation is choosing between a rhythm control strategy or a rate control strategy as the main strategy. When choosing rate control, the optimal heart rate target is uncertain. The Danish Atrial Fibrillation trial is a randomized, multicenter, two-group, superiority trial comparing strict rate control versus lenient rate control in patients with either persistent or permanent atrial fibrillation at inclusion. To prevent bias arising from selective reporting and data-driven analyses, we developed a predefined description of the statistical analysis.

**Methods:**

The primary outcome of this trial is the physical component score of the SF-36 questionnaire. A total of 350 participants will be enrolled based on a minimal important difference of 3 points on the physical component score of the SF-36 questionnaire, a standard deviation of 10 points, a statistical power of 80% (beta of 20%), and an acceptable risk of type I error of 5%. All secondary, exploratory, and echocardiographic outcomes will be hypothesis-generating. The analyses of all outcomes will be based on the intention-to-treat principle. We will analyze continuous outcomes using linear regression adjusting for “site,” type of atrial fibrillation at inclusion (persistent/ permanent), left ventricular ejection fraction (≥ 40% or < 40%), and the baseline value of the outcome (all as fixed effects). We define our threshold for statistical significance as a *p*-value of 0.05 and assessments of clinical significance will be based on the anticipated intervention effects defined in the sample size and power estimations. Thresholds for both statistical and clinical significance will be assessed according to the 5-step procedure proposed by Jakobsen and colleagues.

**Discussion:**

This statistical analysis plan will be published prior to enrolment completion and before any data are available and is sought to increase the validity of the DANish Atrial Fibrillation trial.

**Trial registration:**

Clinicaltrials.gov NCT04542785. Registered on Sept 09, 2020.

**Supplementary Information:**

The online version contains supplementary material available at 10.1186/s13063-023-07247-7.

## Background

Atrial fibrillation is the most common arrhythmia of the heart with a prevalence of approximately 2 to 4% in the western world [1]. A key decision in the treatment of atrial fibrillation is choosing between a rhythm control strategy and a rate control strategy as the main treatment strategy. When choosing rate control, the optimal heart rate target is uncertain [1].

Current guidelines consider lenient rate control acceptable as the initial rate-controlling strategy [1]. This recommendation is primarily based on the results of the RACE II trial [2]. The Danish Atrial Fibrillation (DanAF) trial is a randomized, two-group, multicenter, superiority trial that plans to investigate which heart rate target is superior regarding quality of life in patients with atrial fibrillation [3].

The present publication will summarize the statistical analysis plan to ensure that the trial is analyzed according to a prespecified plan as recommended by The International Conference on Harmonization of Good Clinical Practice, among others, to prevent bias arising from selective reporting and data-driven analyses [4, 5]. The trial is registered with Clinicaltrials.gov: NCT04542785.

## Methods

**Fig. 1:**
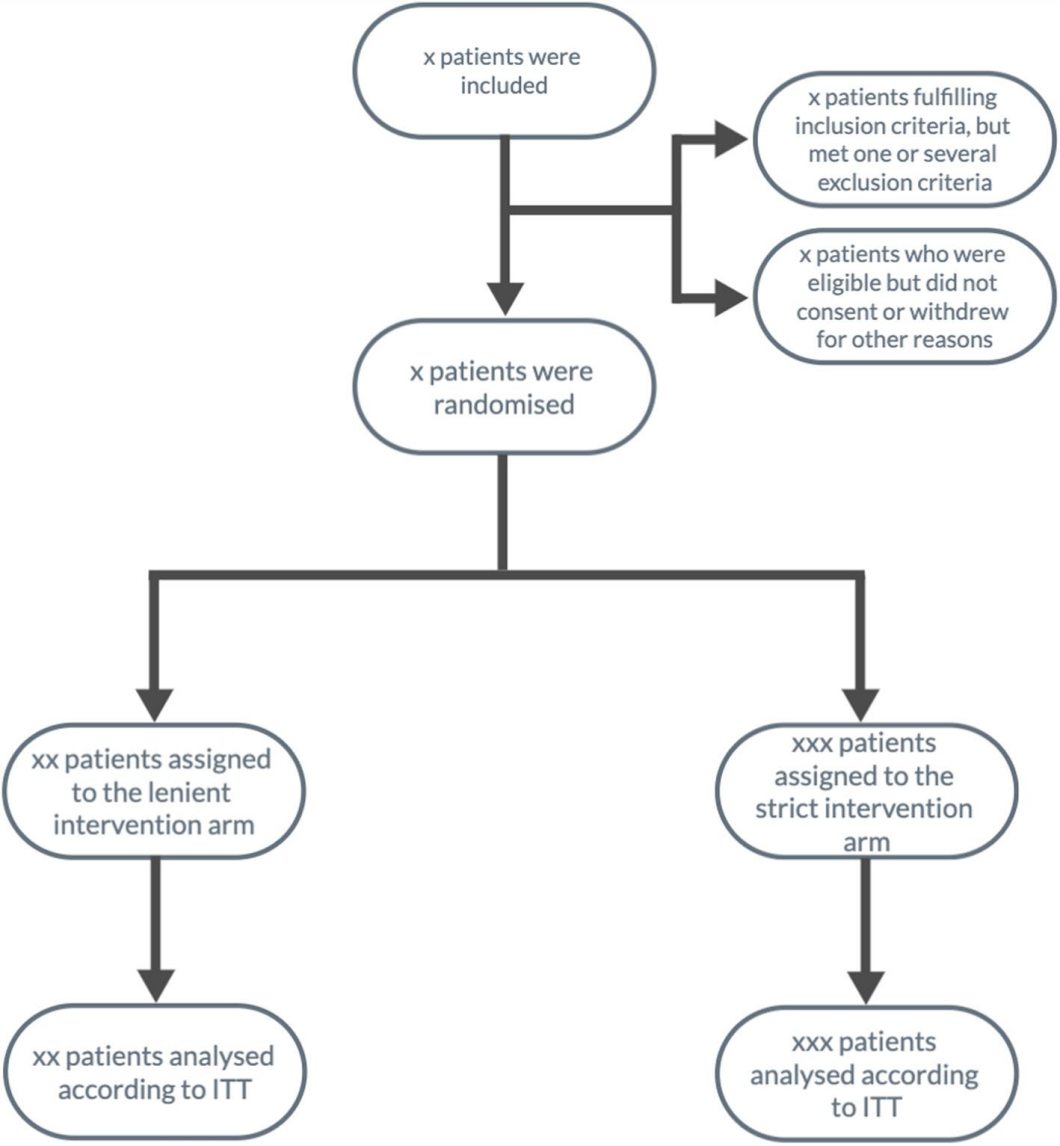
CONSORT flow diagram

The design of this trial has been described in detail in our protocol for this trial [3]. The flow of the recruitment, exclusion, and randomization process is displayed in a Consolidated Standards of Reporting Trials flow diagram (CONSORT) (see Fig. 1) [6].

**Inclusion criteria**

- Participants with either persistent (defined as atrial fibrillation lasting for more than 7 days) or permanent (rate control is considered the only treatment option) atrial fibrillation confirmed by 12-lead electrocardiogram (ECG) at inclusion. Participants with postoperative atrial fibrillation who meet the listed inclusion criteria and none of the exclusion criteria will be eligible as well.
- Rate control must be accepted as the primary management strategy.
- Informed consent.
- Adult (18 years or older).

**Exclusion criteria**

- No informed consent
- Initial heart rate below 80 bpm at rest (assessed by 12-lead ECG before randomization)
- Oral anticoagulant therapy of non-vitamin K oral anticoagulants (NOACs) of less than 3-week duration or warfarin with international normalized ratio (INR) levels within therapeutic range or less than 4 weeks
- If the treating physician deems that the participant is not suitable to be randomized into both groups based on an individual assessment. This decision will be made before randomization by the treating physician. This can, for example, include participants depending on a high ventricular rate to maintain a sufficient cardiac output, such as patients with heart failure, a hemodynamically significant valve dysfunction, or severely dehydrated participants
- Participants who are hemodynamically unstable and therefore require immediate electrical cardioversion

### Randomization

This trial uses centralized randomization at Open Patient data Explorative Network (OPEN), where varying block sizes unknown to the investigators will be used. Randomization is stratified for (1) site of inclusion, (2) persistent/permanent atrial fibrillation (6 months before inclusion), and (3) left ventricular ejection fraction (LVEF) (≥ 40% or < 40%). Participants are be randomly allocated to the lenient versus the strict rate control strategy arm in an 1:1 ratio [3].

### Participant withdrawal

Participants can withdraw his or her consent at any time point for any reason. The participants will be asked if he or she will still participate in the follow-up assessment(s). As treatment follows standard treatment for atrial fibrillation and any treatment clinically indicated is accepted, there are no other reasons that will result in participants being withdrawn [3].

### Trial interventions

It is up to the clinician to treat the patient with the guideline-appropriate heart rate-reducing agents [1]. This is further explained in the protocol of this trial, and the European Society of Cardiology guidelines, who list the suggested agents and doses to achieve rate control [1, 3].

#### Lenient rate control

For lenient rate control, the treatment provider will target the highest tolerable resting heart rate < 110 bpm assessed on a 12-lead resting ECG measured over 1 min after 5 min of rest. If the heart rate is below 90 beats per minute, the responsible physician is encouraged to reduce rate-controlling drugs.

#### Strict rate control

For strict rate control, the treatment provider will target a mean resting heart rate of 70 bpm, but < 80 bpm is acceptable. The heart will be assessed on a 12-lead resting ECG measured over 1 min after 5 min of rest.

A more detailed description of the interventions can be found in the protocol, which previously has been published [3].

### Outcomes

We want to assess all relevant outcomes. To limit problems with multiplicity, we define outcome hierarchies. The outcomes are defined as primary, secondary, exploratory, and echocardiographic outcomes. The sample size (see “Sample size” section) is based on the primary outcome and our primary conclusions will be based on the results of the primary outcome. The results from secondary, exploratory, and echocardiographic outcomes will be considered hypothesis-generating only.

#### Primary outcome

- Quality of life using the SF-36 questionnaire (physical component score)

#### Secondary outcomes

- Hospital-free days (HFDs), analyzed as count data. We define HFDs as all days alive that are spent outside an acute-care hospital, long-term acute-care hospital (LTACH), or in an emergency department (ED), including days spent wholly or in part under “observation” status. All other days, including days spent in a long- or short-stay nursing facility, inpatient hospice facility, or rehabilitation facility count as hospital-free, as would all days at home, including those with home-based medical services [7]
- Symptoms due to atrial fibrillation assessed using the Atrial Fibrillation Effect on Quality-of-Life questionnaire
- Quality of life using the SF-36 questionnaire (mental component score)
- Serious adverse events, defined according to The International Conference on Harmonization as any untoward medical occurrence that resulted in death, was life-threatening, required hospitalization or prolongation of existing hospitalization, and resulted in persistent or significant disability or jeopardized the patient [4]

#### Exploratory outcomes

- All-cause mortality
- Composite of all-cause mortality, stroke, myocardial infarction, and cardiac arrest
- Stroke
- Hospitalization for worsening of heart failure
- Number of hospital admissions, analyzed as count data
- Six-minute walking distance
- Healthcare costs (will be further defined and reported in separate publications)
- Various biomarkers (N-terminal pro-brain natriuretic peptide (nt-proBNP), high-sensitivity C reactive protein (hsCRP), high-sensitivity troponin I (hsTnI), growth differentiation factor-15 (GDF-15), interleukin 6 (IL6), cystatin-C, YKL40, soluble urokinase plasminogen activator receptor (suPAR), and fibulin-1
- Switch to rhythm control strategy (such as rhythm control medication, direct current-conversion or pharmacological cardioversion, pulmonary vein isolation, or arrhythmia surgery)
- Implantation of a pacemaker or cardioverter-defibrillator with or without atrioventricular nodal ablation

#### Echocardiographic outcomes

A detailed description of the echocardiographic analysis principles can be found in supplemental file 1. Additionally, we will develop and publish a separate echocardiographic statistical analysis plan.

The following outcomes will be assessed in a core-echo lab:

- Left atrial size (left atrial volume index)
- Left ventricular size
- Cardiac index (cardiac output/body surface area)
- Left ventricular ejection fraction
- Tricuspid annular plane systolic excursion (TAPSE)
- Midwall fractional shortening
- Global longitudinal strain
- Circumferential end-systolic stress
- Diastolic dysfunction estimated by the relationship between left ventricular filling and the interval between two successive R waves on ECG (R-R interval) for the individual patient
- Pulmonary pressure

#### Subgroup analysis

The subgroup analyses will be performed for primary and secondary outcomes and all subgroup analyses will be regarded as hypothesis-generating only. These analyses will be tested for interactions using test of interaction (interaction between the treatment variable and the subgroup indicator) in STATA 17 [8].

We will compare the following subgroups between the intervention arms:

- Patients with heart failure (including subtypes) compared to patients without heart failure [9]
  - ◦ Heart failure with reduced ejection fraction LVEF ≤ 40% or mildly reduced ejection fraction LVEF 41–49% (HFrEF and HFmrEF)
  - ◦ Heart failure with preserved ejection fraction LVEF ≥ 50% (HFpEF)
- Patients compared by their New York Heart Association (NYHA) class
  - ◦ NYHA class: I
  - ◦ NYHA class: II
  - ◦ NYHA class: III and IV
- Patients who are men compared to patients who are women
- Patients compared based on their different durations of atrial fibrillation at randomization. The starting point of the duration of atrial fibrillation will be defined by when the patient was clinically diagnosed with persistent atrial fibrillation
  - ◦ Less than 1 year
  - ◦ 1 to 2 years
  - ◦ More than 2 years
- Patients who are 75 years of age or older compared to patients below 75 years of age
- Patients according to the modified European Heart Rhythm Association (mEHRA) symptoms score [10]
  - ◦ mEHRA score: 1, 2a, and 2b
  - ◦ mEHRA score: 3 and 4
- Patients having persistent atrial fibrillation compared to patients having permanent atrial fibrillation. Persistent atrial fibrillation defined as atrial fibrillation for more than 7 days and permanent atrial fibrillation defined as where only rate control is considered going forward
- Patients achieving the target heart rate compared to patients not achieving the target heart rate

### Sample size

We estimated the required sample to be a total of 350 participants based on a minimal important difference of 3 points on the physical component score of the SF-36 questionnaire, a standard deviation (SD) of 10 points, power of 80%, and an acceptable risk of type I error of 5% [3].

#### Power estimations of secondary and exploratory outcomes

All power estimations below are based on the sample size estimation of 350 participants. The remaining power estimations can be found in supplemental file 2.

#### Hospital-free days

Using a minimal important difference of 3 days, a SD of 9 days, a risk of type I error of 5%, and accounting for the fact that the data is expected not to be normally distributed (adding 15% to the required sample size) [11], we will be able to reject the null hypothesis that the population means of the experimental and control groups are equal with probability (power) of 82.1% [12].

#### The Atrial Fibrillation Effect on Quality-of-Life

Using a minimal important difference of 7 points, a SD of 21 points, and a risk of type I error of 5%, we will be able to reject the null hypothesis that the population means of the experimental and control groups are equal with probability (power) of 87.5% [13, 14].

#### Quality of life using the SF-36 questionnaire (mental component score)

Using a minimal important difference of 4, a SD of 10, and a risk of type I error of 5%, we will be able to reject the null hypothesis that the population means of the experimental and control groups are equal with probability (power) of 96% [15–17].

#### Serious adverse events

Using a proportion of participants with one or more serious adverse events in the control group of 20%, a relative risk reduction of 30%, and a risk of type I error of 5%, we will be able to reject the null hypothesis with probability (power) of 32%.

### Timeframe of analyses and investigations

Investigations of this trial will take place at the baseline visit, followed by a 1-month, 2-month, 6-month, and 12/24/36-month visits. A margin of 2 weeks + / − of each visit will be allowed. Further visits might be needed to achieve the desired rate. A more detailed description of the timeframe of investigations can be found in Table 2 of the protocol [3].

The primary analyses of all outcomes of this trial will be performed after all participants have completed a 12-month follow-up (after randomization). The 12-month follow-up will be regarded as our primary analysis timepoint, and the 24-month and 36-month analyses will be considered hypothesis-generating only to limit multiplicity.

Furthermore, an independent data safety monitoring committee (IDSMC) will conduct an interim analysis after 33% of the sample size population has completed 12-month follow-up, to monitor whether the trial still holds scientific merit. The IDSMC will then decide if a new interim analysis should be performed.

A more detailed description of the IDSMC and its interim analyses can be found in the protocol and the supplemental file 6 of the protocol [3].

### General analytic principles

The analyses of the outcomes will be based on the intention-to-treat principle, meaning that all participants will be analyzed in the group they were randomized to. For each group, we will report the proportion of participants who are randomized, the ones who receive a rhythm control strategy, and those who do not achieve the allocated heart rate target and the reason why.

We define our threshold for statistical significance as a *p*-value of 0.05, and assessments of clinical significance will be based on the anticipated intervention effects defined in the sample size and power estimations. Thresholds for both statistical and clinical significance will be assessed according to the 5-step procedure proposed by Jakobsen and colleagues [18].

We will adjust all regression analyses for the stratification variables we used for the randomization process and continuous outcomes will additionally be adjusted for the baseline value of the variable [19–21].

#### Statistical analyses

The statistical analyses will be performed using STATA 17 [8].

##### Analysis of continuous data

Continuous outcomes will be presented as means and SD with 95% confidence intervals (Cis). We will analyze continuous outcomes using linear regression adjusting for “site,” type of atrial fibrillation (persistent/ permanent), LVEF (≥ 40% or < 40%), and the baseline value of the score (all as fixed effects) [22].

In the quality-of-life analysis, our primary analyses will only be of participants who are alive and able to fill out the questionnaires at the 12-month follow-up will be included. If a participant has died, he/she will not be included in this analysis. To assess the potential influence of participants who die, we will present a sensitivity analysis as a supplement where participants who die will have a value of 0 imputed for quality of life (these data will be analyzed as count data, see paragraph below).

An example of continuous data is collected from the following outcome: “SF-36 questionnaire score (physical component).”

##### Analysis of dichotomous data

Dichotomous outcomes will be presented as proportions of participants in each group with the event, as well as relative risks with 95% CIs. Dichotomous outcomes will be analyzed using logistic regression adjusting for “site,” type of atrial fibrillation at inclusion (persistent/permanent), and LVEF (≥ 40% or < 40%) (all as fixed effects). Odds ratios will be transformed to relative risks using the NLCOM command in STATA [8].

An example of a dichotomous data is collected from the following outcome: “Switch to rhythm control therapy.”

##### Analysis of count data

Count data will be presented as medians and interquartile ranges. We will analyze count data using van Elteren’s test stratifying for “site” and report Hodges Lehman median differences and confidence intervals [12, 23, 24].

An example of a count outcome is collected from the following outcome: “Number of hospital admissions.”

#### Baseline characteristics

The baseline characteristics which will be reported for both treatment arms separately are shown in Table 1 [25]. We will not undertake any formal test of comparison between the two groups. For continuous data, we will present mean and standard deviation if normally distributed, or median and interquartile range if the data is skewed. Categorical data will be presented with absolute numbers and percentages.

**Table 1:** Baseline characteristics

Characteristic	Lenient rate control (*N* = X)	Strict rate control (*N*= X)
General characteristics		
Age, years		
Female sex–no. (%)		
Site of recruitment		
Holbæk		
Roskilde		
Odense		
Bispebjerg		
Hvidovre		
Duration of atrial fibrillation–weeks		
Median(IQR)		
Previous electrical cardioversion–no. (%)		
Previous valvular heart disease, valvular surgery, or valvular replacement–no. (%)		
Comorbidities		
CHA_2_DS_2_-VASc score–no. (%) [16]^a^		
0 (low)		
1 (low-moderate)		
2 or greater (highest: 9) (moderate-high)		
Diabetes mellitus – no. (%)		
Hypertension – no. (%)		
Chronic obstructive lung disease – no. (%)		
Coronary artery disease – no. (%)		
Valvular heart disease – no. (%)		
Chronic heart failure		
Systolic heart failure – no. (%)		
Diastolic heart failure – no. (%)		
NYHA classification – no. (%)		
Class I		
Class II		
Class III		
Class IV		
Number of heart failure hospitalizations – no. (%)		
Alcohol consumption – no. /week		
Tobacco pack years – no		
Pacemaker or implantable cardioverter-defibrillator – no. (%)		
Symptoms – no. (%)		
Dyspnea		
Fatigue		
Palpitations		
mEHRA score – no. (%)		
Score: 1		
Score: 2a		
Score: 2b		
Score: 3		
Score: 4		
General characteristics		
BMI		
Blood pressure – mm Hg		
Systolic		
Diastolic		
Heart rate at rest – BMP		
Rate control medications in use at baseline – no. (%)		
None		
Metoprolol		
Atenolol		
Bisoprotol		
Carvedilol		
Digoxin		
Digoxin and beta-blocker		
Verapamil		
Verapamil and beta-blocker		
Amiodarone		
Other medications in use – no. (%)		
Diuretic		
Anticoagulants		
Antiplatelets		
Glucagon-like peptide 1 receptor agonists		
Sodium-glucose cotransporter-2 inhibitors		
Angiotensin-converting enzyme inhibitors		
Angiotensin receptor blockers		

^a^Congestive heart failure, hypertension, age ≥ 75 (doubled), diabetes, stroke (doubled), vascular disease, age 65 to 74, and sex category (female). The CHA_2_DS_2_-VASc score is a risk model for anticoagulation decision-making in atrial fibrillation patients [22]

#### Handling of missing data

Missing data will be handled according to the recommendations proposed by Jakobsen and colleagues [22]. In short, we will investigate the possible pattern of missing data. If it is plausible that data are missing at random, and the amount of missing data is between 5 and 40%, we will use multiple imputations as a secondary analysis [22]. If less than 5% of data are missing, we will only use participants with follow-up data.

If necessary, we will perform a best-worst- and a worst-best-case scenario. These two scenarios demonstrate the maximum potential impact of missing data. When assessing continuous data, a “beneficial” outcome will be defined as plus two SDs of the group mean, and a “harmful” outcome defined as minus two SDs of the group mean, both “beneficial” and “harmful” outcomes being fixed imputations [22].

When assessing dichotomous data, it will in a best-worst case scenario be assumed that all the participants lost to follow-up in the lenient intervention arm have had a “beneficial” outcome and all the participants lost to follow-up in the strict intervention arm have had a “harmful” outcome [22], conversely, for the worst-best-case scenario [22].

#### Assessments of underlying statistical assumptions

We will systematically assess underlying statistical assumptions for all statistical analyses [26, 27]. For all regression analyses, both primary and secondary, we will test for major interactions between each covariate and the intervention variable. When assessing for major interactions, we will, in turn, include each possible first-order interaction between included covariates and the intervention variable [26, 27]. For each combination, we will test if the interaction term is significant and assess the effect size. We will only consider that there is evidence of an interaction if the test of interaction is statistically significant after Bonferroni adjusted thresholds (0.05 divided by number of possible interactions (treatment variable interaction with “site,” persistent/permanent, and LVEF (≥ 40% or < 40%) = 0.017)) [26, 27]. If it is concluded that the test of interaction is significant, we will consider both presenting an analysis separately for each site (e.g., for each site if there is significant interaction between the trial intervention and “site”) and an overall analysis including the interaction term in the model [26, 27].

#### Assessments of underlying statistical assumptions for linear regression

We will visually inspect quantile–quantile plots of the residuals [28, 29] to assess if the residuals are normally distributed and use residuals plotted against covariates and fitted values [28, 29] to assess for homogeneity of variances [28, 29]. If the plots show deviations from the model assumptions, we will consider transforming the outcome, e.g., using log transformation or square root and/or use robust standard errors [26, 28, 29].

#### Assessments of underlying statistical assumptions for dichotomous outcomes

We will assess if the deviance divided by the degrees of freedom is significantly larger than 1 to assess for relevant overdispersion. Overdispersion is the presence of greater variability (statistical dispersion) in a data set than would be expected based on a given statistical model, and this case considered using a maximum likelihood estimate of the dispersion parameter [27]. We will, by checking if the number of events is larger than 10 (rule of thumb) per site, consider pooling the data from smaller sites if the number of events is too low [27].

#### Statistical reports

Blinded data will be sent to OPEN for blinded data management [3]. Statistical analyses will be performed with the two intervention groups coded as “A” and “B” by two independent blinded statisticians [3]. Two blinded conclusions will be drawn by the steering group: one assuming “A” is the experimental group and “B” is the control group—and one assuming the opposite. Based on these two blinded conclusions, two abstracts will be written (will be published as a supplement to the main publication) [3]. When the blinding is broken, the “correct” abstract will be chosen, and the conclusions in this abstract will not be revised [3].

## Discussion

To prevent bias arising from selective reporting and data-driven analyses, we present this pre-defined description of the statistical analysis plan of the DanAF trial.

### Strengths

Our analysis plan has several strengths. We have predefined our analysis plan and this will reduce outcome reporting bias and data-driven results. Our conclusions will be based on only one primary outcome, i.e., quality of life (measured using SF-36 physical component score) which is a patient-important outcome and is therefore of high relevance to the patients and the clinicians when choosing treatment. Our sample size estimation is based on similar studies assessing the quality of life and studies assessing minimal important differences [15–17]. Our other secondary, exploratory, and echocardiographic outcomes will be hypothesis-generating only; hence, problems regarding multiplicity will be limited (see the “Limitations” section). The inclusion and exclusion criteria of this trial are few, which should increase the external validity of our trial.

We have performed a sample size estimation based on previous evidence [15–17]. With realistic intervention effects, we will adjust the thresholds for statistical significance and the confidence intervals if the sample size is not reached [3]. In Denmark, a complete follow-up of all participants for death and hospitalizations is possible, as all residents are issued a permanent unique civil registration number at birth or immigration that enables individual-level linkage between administrative registries [3]. If necessary, we will use multiple imputation and best–worst/worst-best-case scenarios to assess the potential impact of the missing data on the results [22]. Furthermore, we plan to systematically assess whether underlying statistical assumptions are fulfilled for all statistical analyses.

Hence, our trial will be conducted with low risks of both random errors (“play of chance”) and systematic errors (“bias”) [3, 18, 30].

### Limitations

Our analysis plan also has limitations. According to our power estimations, the number of recruited participants will most likely not allow us to conclude on outcomes such as mortality or serious adverse events [3]. Therefore, even if one of the interventions turns out to be superior in terms of quality of life, it will be uncertain how the trial interventions influence hard outcomes such as, e.g., death. This will be explored in a future meta-analysis with individual patient data from the RACE II trial [16]. The consequence may ultimately be that a superiority trial in terms of “hard outcomes” is needed [3].

The results of the EAST trial [31] are expected to delay when rhythm control is abandoned for rate control only. This may impact on the generalizability of our results as some participants who are now included in DanAF may in the future instead be treated with rhythm control [3].

Yet another limitation is that participants presumably will receive different medications and procedures in the compared groups [3]. If we show a difference (or lack of a difference) between the groups, it will be difficult to interpret what part of the treatment algorithm for reaching a certain rate target that caused this difference [3].

## Supplementary Information

**Additional file 1.** Echocardiographic analysis principles.

**Additional file 2.** Power estimations of exploratory outcomes.

## Data Availability

Region Zealand is the data controller. Steering committee members have access to all data. Anonymized data will be made available in a file repository. All data regarding the statistical analysis plan is available in the present publication.
