# Supplementary material for: Lenient rate control versus strict rate control for atrial fibrillation: a statistical analysis plan for the Danish Atrial Fibrillation (DanAF) randomized clinical trial
Source: Trials. 2023 Apr 1;24:250. doi: 10.1186/s13063-023-07247-7 (PMC10068144; doi:10.1186/s13063-023-07247-7)
Supplement: Supplementary file 1 — Additional file 1. Echocardiographic analysis principles. [file 13063_2023_7247_MOESM1_ESM.docx]

**DanAF**

**Echocardiographic analyses plan**

**AORTA**

Aorta sinus _ _ mm ASI x x x mm/m^2^

Aorta ascendens _ _ mm AAI x x x mm/m^2^

**Left ventricle dimensions**

LVIDd: _ _ mm LVIDId x x x mm/m^2^

IVSd: _ _ mm IVSId: x x x mm/m^2^

LVPWd: _ _ mm LVPWId: x x x mm/m^2^

LVIDs: _ _ mm LVIDIs: x x x mm/m^2^

IVSs: _ _ mm

LVPWs: _ _ mm

FS: x x % (LV endocardial shortening) FS = (LVIDd - LVIDd) / LVIDd

LVM_a-I_: _ _ _ g (2D areal-length formel)

LVMI_a-I_: x x x g/m^2^

RWT: x , x x = (2 x LVPWd) /LVIDd

Longitudinal strain profile

LVGLS: _ _, _ %

LV volume and systolic function (Simpson method):

LVEDV_2D plani_: _ _ _ ml

LVESV_2D plani_: _ _ _ ml

HR_Simpson_ _ _ _ bpm

LVSV_2D plani_ x x ml = LVEDV_2D plani_ – LVESV_2D plani_

LVSVI_2D plani_ x x ml/m^2^

LVCO_2D plani_ x, x x l = LVSV_2D plani_ x HR_Simpson_

LVCI_2D plani_ x, x x l/m^2^

LVEF_2D plani_ x x % = (LVEDV_2D plani_ – LVESV_2D plani_) / LVEDV_2D plani_

LVSV and LVCO using doppler

LVOT: _ _ mm

VTI_LVOT_ _ _ , _ cm

HR_SV,LVOT_: _ _ _ bpm

LVSV_Doppler_ x x ml = π x r^2^_LVOT_ x VTI_LVOT_ = 3,146 x (LVOT/2)^2^ x VTI_LVOT_

LVSVI_Doppler_ x x ml/m^2^

LVCO_Doppler_ x , x x l = LVSV_Doppler_ x HR_SV,LVOT_

LVCI_Doppler_ x , x x l/m^2^

LVSV og LVCO calculated from 10 consecutive cycles:

LVOT: _ _ mm

VTI_LVOT_ _ _ , _ cm

HR_SV,LVOT_: _ _ _ bpm

LVSV_Doppler_ x x ml = π x r^2^_LVOT_ x VTI_LVOT_ = 3,146 x (LVOT/2)^2^ x VTI_LVOT_

LVSVI_Doppler_ x x ml/m^2^

LVCO_Doppler_ x , x x l = LVSV_Doppler_ x HR_SV,LVOT_

LVCI_Doppler_ x , x x l/m^2^

LV diastolic function

E’ lat: _ _ , _ cm/s

E’ med: _ _ , _ cm/s

E: _ _ _ cm/s

DT: _ _ _ msec

IVRT _ _ _ msec

E/E’lat: x x , x

E/E’med: x x , x

**Left atrium**

LA diam: _ _ mm

LAEDV: _ _ _ ml LAEDVI: x x ml/m^2^

LAESV: _ _ _ ml LAESVI: x x ml/m^2^

**Right ventricle**

RVD1: _ _ mm RVD1I: x x mm/m^2^

RVD2: _ _ mm RVD2I: x x mm/m^2^

TAPSE: _ _ mm

RVA_D_: _ _, _ cm^2^ RVA_D_I: xx,x cm^2^/m^2^

RVA_S_: _ _ , _ cm^2^ RVA_S_I: xx ,x cm^2^/m^2^

RV FAC: x x %

**Right atrium**

RAarea: _ _ , _ cm^2^

RAareaI: _ _ , _ cm^2^/m^2^

**Pulmonary pressure**

AT_RVOT_: _ _ _ msec

MPAP: x x mmHg = 80 – (0,5 x AT_RVOT_)

TR max PG: _ _ mmHg

PAP_peaksyst_: _ _ mmHg

**Additional findings**

**Abbreviations.**

LVIDd – Left ventricular internal diameter at end-diastole

LVIDId – Left ventricular internal diameter index at end-diastole

IVSd – Intraventricular septum thickness at end-diastole

IVSId - Intraventricular septum thickness index at end-diastole

LVPWd – Left ventricular posterior wall thickness at end-diastole

LVPWId - Left ventricular posterior wall thickness index at end-diastole

IVSs – Intraventricular septum thickness at end-systole

LVPWs – Left ventricular posterior wall thickness at end-systole

FS – Fractional shortening

LVM – Left ventricular mass

LVMI – Left ventricular mass index

RWT – Relative wall thickness

LVGLS – Left ventricular global longitudinal strain

LVEDV – Left ventricular end-diastolic volume

LVESV – Left ventricular end-systolic volume

HR – Heart rate

LVSV – Left ventricular stroke volume

LVSVI – Left ventricular stroke volume index

LVCO – Left ventricular cardiac output

LVCI – Left ventricular cardiac output index

LVEF – Left ventricular ejection fraction

LVOT – Left ventricular outflow tract

VTI – Velocity time integral

E’ lat - Peak velocity of early diastolic lateral mitral annular motion as determined by pulsed wave Doppler

E’ med - Peak velocity of early diastolic medial mitral annular motion as determined by pulsed wave Doppler

E - Peak velocity of early diastolic transmitral flow

DT – Deceleration time of early diastolic transmitral flow

IVRT – Isovolumetric relaxation time

LA – Left atrium

LAEDV – Left atrial end-diastolic volume

LAESV – Left atrial end-systolic volume

RVD – Right ventricular diameter

TAPSE - Tricuspid Annular Plane Systolic Excursion

RVA – Right ventricular area

RV FAC – Right ventricular fraction area change

RA – Right atrium

AT – Acceleration time

RVOT – Right ventricular outflow tract

MPAP – Main pulmonary artery pressure

TR PG – Tricuspid regurgitation max pressure gradient

PAP – Pulmonary artery pressure
