# Supplementary material for: Lenient rate control versus strict rate control for atrial fibrillation: a statistical analysis plan for the Danish Atrial Fibrillation (DanAF) randomized clinical trial
Source: Trials. 2023 Apr 1;24:250. doi: 10.1186/s13063-023-07247-7 (PMC10068144; doi:10.1186/s13063-023-07247-7)
Supplement: Supplementary file 2 — Additional file 2. Power estimations of exploratory outcomes. [file 13063_2023_7247_MOESM2_ESM.docx]

**Supplemental file 2 – Power estimations of exploratory outcomes**

The below power calculations are based on a sample size of 350 participants as specified in the main document.

**POWER ESTIMATIONS OF EXPLORATORY OUTCOMES**

**All-cause mortality**

Using a proportion of participants dying (all-cause mortality) in the control group of 5%, a relative risk reduction of 10%, and a risk of type I error of 5% we will be able to reject the null hypothesis with probability (power) of 5.7% [1].

**Composite of all-cause mortality, stroke, myocardial infarction, and cardiac arrest**

Using a proportion of participants either dying (all-cause mortality), or experiencing stroke, myocardial infarction, or cardiac arrest in the control group of 8.0%, a relative risk reduction of 10%, and a risk of type I error of 5% we will be able to reject the null hypothesis with probability (power) of 5.9% [1, 2].

**Cardiac mortality**

Using a proportion of participants dying from cardiac events in the control group of 3.9%, a relative risk reduction of 10%, and a risk of type I error of 5% we will be able to reject the null hypothesis with probability (power) of 5.4% [1].

**Stroke**

Using a proportion of participants experiencing a stroke in the control group of 3.9%*, a relative risk reduction of 10%, and a risk of type I error of 5% we will be able to reject the null hypothesis with probability (power) of 5.4% [1].

**Hospitalization for worsening of heart failure**

Using a proportion of participants experiencing hospitalization due to worsening of heart failure in the control group of 27,4%, a relative risk reduction of 10%, and a risk of type I error of 5% we will be able to reject the null hypothesis with probability (power) of 9.0% [1].

**Number of hospital admissions**

Using a proportion of participants who are hospitalized in the control group of 27,4%, a relative risk reduction of 10%, and a risk of type I error of 5% we will be able to reject the null hypothesis with probability (power) of 9% [1].

**Six-minute walking distance**

Using a minimal important difference of 40, a SD of 75, and a risk of type I error of 5% we will be able to reject the null hypothesis that the population means of the experimental and control groups are equal with probability (power) of 99,9% [3-5].

**Physical activity using trial accelerometer - sedentary behavior**

Using a minimal important difference of 20 minutes per day, a SD of 65 minutes per day, and a risk of type I error of 5% we will be able to reject the null hypothesis that the population means of the experimental and control groups are equal with probability (power) of 81,9% [6, 7].

**: Van Gelder and colleagues states that the incidence of a stroke occurring in the strict rate control group is 3,9%. However, adding up the ‘Ischemic’-stroke and ‘Hemorrhagic’-stroke colons equals 12 participants, and not 11 participants as stated. The percentage of this event could therefore be 4,4% instead of 3,9% as stated.*

**References**

1. Van Gelder, I.C., et al., *Lenient versus Strict Rate Control in Patients with Atrial Fibrillation.* New England Journal of Medicine, 2010. **362**(15): p. 1363-1373.

2. Kirchhof, P., et al., *Improving outcomes in patients with atrial fibrillation: rationale and design of the Early treatment of Atrial fibrillation for Stroke prevention Trial.* Am Heart J, 2013. **166**(3): p. 442-8.

3. Andrea, P., et al., *Short-Term Change in Distance Walked in 6 Min Is an Indicator of Outcome in Patients With Chronic Heart Failure in Clinical Practice.* Journal of the American College of Cardiology, 2006. **48**(1): p. 99-105.

4. Silvet, H., L.A. Hawkins, and A.K. Jacobson, *Heart Rate Control in Patients With Chronic Atrial Fibrillation and Heart Failure.* Congestive Heart Failure, 2013. **19**(1): p. 25-28.

5. Ding, L., et al., *Correlation between impedance cardiography and 6 min walk distance in atrial fibrillation patients.* BMC Cardiovascular Disorders, 2016. **16**(1): p. 133.

6. Bellettiere, J., et al., *Sedentary behavior and cardiovascular disease in older women: The Objective Physical Activity and Cardiovascular Health (OPACH) Study.* Circulation, 2019. **139**(8): p. 1036-1046.

7. Andersson, C., et al., *Physical activity measured by accelerometry and its associations with cardiac structure and vascular function in young and middle-aged adults.* J Am Heart Assoc, 2015. **4**(3): p. e001528.
